# Formation of vesicles-in-a-vesicle with asymmetric lipid components using a pulsed-jet flow method

**DOI:** 10.1039/c9ra04622d

**Published:** 2019-09-24

**Authors:** Koki Kamiya, Toshihisa Osaki, Shoji Takeuchi

**Affiliations:** Artificial Cell Membrane Systems Group, Kanagawa Institute of Industrial Science and Technology 3-2-1 Sakado, Takatsu-ku Kawasaki Kanagawa 213-0012 Japan; Department of Mechano-Informatics, Graduate School of Information Science and Technology, The University of Tokyo 7-3-1 Hongo, Bunkyo-ku Tokyo 113-8656 Japan takeuchi@hybrid.t.u-tokyo.ac.jp; Division of Molecular Science, Graduate School of Science and Technology, Gunma University 1-5-1 Tenjin-cho Kiryu Gunma 376-8515 Japan

## Abstract

Lipid distribution in intracellular vesicles is different from that in the plasma membrane of eukaryotic cells. The lipid components in the intracellular vesicles are composed of phosphatidylserine and phosphatidylethanolamine in the outer leaflet and phosphatidylcholine and sphingomyelin in the inner leaflet. The lipid asymmetricities both in the intracellular vesicle membrane and the plasma membrane contribute to synaptic transmission functions. In this study, we developed a cell-sized asymmetric lipid vesicle system containing small-sized asymmetric lipid vesicles (of diameter 200–1000 nm) (asymmetric vesicles-in-a-vesicle), emulating lipid components in the plasma membrane and intracellular vesicle membrane of eukaryotic cells, using microfluidic technology. We successfully constructed an artificial exocytosis system using the asymmetric vesicles-in-a-vesicle system. This asymmetric vesicles-in-a-vesicle system will be helpful in understanding the mechanisms of vesicle transport, such as neurotransmission and exocytosis.

## Abstract 2

We develop a cell-sized asymmetric lipid vesicle system containing small-sized asymmetric lipid vesicles using microfluidic technology.

## Graphical abstract

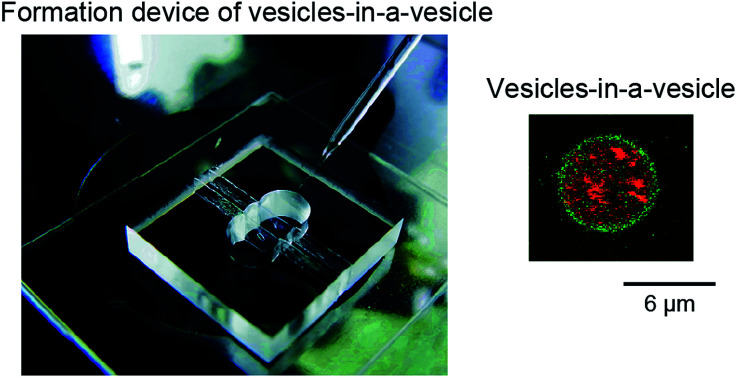

## Introduction

The distribution of lipids in the cell membrane of eukaryotes is asymmetric.^1–4^ The intracellular vesicles play roles in the regulation of metabolism and homeostasis *via* extracellular transport of proteins, ions, and small biological molecules.^5,6^ The intracellular vesicles have an asymmetric lipid bilayer, composed of phosphatidylserine (PS) and phosphatidylethanolamine (PE) in the outer leaflet and phosphatidylcholine (PC) and sphingomyelin in the inner leaflet.^7^ Lipid distribution in the intracellular vesicles is different from that in the plasma membrane. The asymmetricities in lipid distribution both in the intracellular vesicle membrane and the plasma membrane contribute to synaptic transmission functions.^8^ To create an *in vitro* model of this synaptic system using an artificial lipid vesicle system, cell-sized lipid vesicles containing small vesicles (vesicles-in-a-vesicle) have been developed.^9,10^ The vesicles-in-a-vesicle system has various functions, such as sequential enzyme cascades and cargo release activity.^11,12^ However, the composition of lipid membrane in these vesicles-in-a-vesicle systems is symmetric. Therefore, a vesicles-in-a-vesicle system with asymmetric membrane composition is needed to better-understand the role of asymmetric lipid distribution in the synaptic system.

**Fig. 1:**
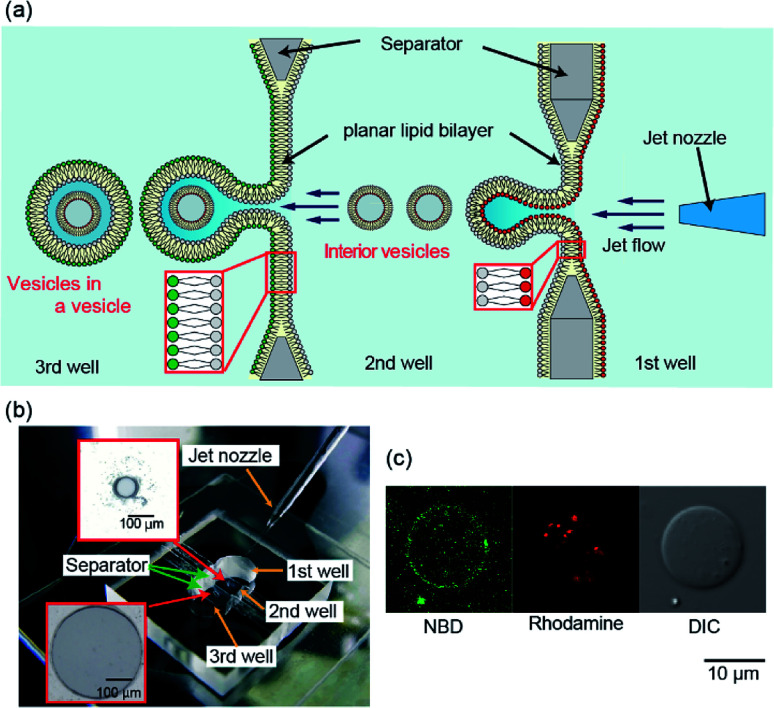
(a) Schematic representation of generation of vesicles-in-a-vesicle system. (b) Image of the fabricated device for generating vesicles-in-a-vesicle system. Separators in this device had apertures of the following two sizes: 100 μm in diameter between the first and second wells and 500 μm in diameter between the second and third wells. (c) A typical confocal image of the vesicles-in-a-vesicle system containing 7-nitro-2-1,3-benzoxadiazol-4-yl (NBD)-lipids in giant vesicle membranes and rhodamine-lipids in the small vesicle.

In this study, using an improved pulsed jet-flow method,^13,14^ we directly prepare cell-sized asymmetric lipid vesicles containing asymmetric lipid vesicles (asymmetric vesicles-in-a-vesicle) to emulate asymmetric lipid distribution in the plasma membrane and intracellular vesicle membrane of eukaryotic cells (Fig. 1(a)). The cell-sized vesicles containing vesicles are generated by applying pulsed jet flow against two parallel planar asymmetric lipid bilayers formed in a triple-well device. First, we optimize the width of the second well in the triple-well device and the application time of the jet flow to enclose the small vesicles within the cell-sized lipid vesicles, with two planar lipid bilayers. Next, the asymmetry of the cell-sized lipid vesicles containing the vesicles generated using the triple-well device is examined by confocal laser scanning microscopy (CLSM). Finally, using the asymmetric vesicles-in-a-vesicle system, we demonstrate the fusion of the inner vesicles with the giant vesicles by introducing calcium ions using biological nanopores.

## Experimental

### Reagents

1,2-Dioleoyl-*sn-glycero*-3-phosphocholine (DOPC), 1,2-dioleoyl-*sn-glycero*-3-phospho-l-serine (DOPS), 1,2-dioleoyl-*sn-glycero*-3-phosphoethanolamine (DOPE), 1,2-dioleoyl-*sn-glycero*-3-phosphoethanolamine-*N*-(lissamine rhodamine B sulfonyl) (Rh-DOPE), and 7-nitro-2-1,3-benzoxadiazol-4-yl (NBD)–DOPE were purchased from Avanti Polar Lipids, Inc. (Alabaster, AL, USA). Annexin V (Alexa Fluor 488-conjugated) was purchased from Thermo Fisher Scientific (Waltham, MA, USA). α-Hemolysin from *Staphylococcus aureus* was purchased from Sigma-Aldrich Co. LLC (St. Louis, MO, USA).

### Device fabrication

The triple-well device for the generation of vesicles-in-a-vesicle system was fabricated by assembling a poly(methyl methacrylate) (PMMA) plate (Acrylight; Mitsubishi Rayon Co., Ltd., Tokyo, Japan) using an automated computer-aided design and computer-aided manufacturing (CAD/CAM) modeling machine (Model MM-100; Modia Systems Co., Inc., Koshigaya, Japan). Well separators, each with one hole of 100 or 500 μm diameter, were prepared from a thin (75 μm) PMMA film and a separator with a single hole was mounted between the wells. The chamber was then attached to a flat, 1 mm-thick acrylic substrate by thermocompression bonding. A micro-jet nozzle with a 60 μm inner-diameter orifice was formed using a thin-walled glass capillary (G-100; Narishige, Tokyo, Japan) with 1 mm outer diameter and a micropipette puller (PC-10; Narishige); the nozzle tip was curved 30° using a microforge (MF-900; Narishige).

### Formation of the vesicles-in-a-vesicle system

Phospholipids (DOPC, DOPS, DOPE, Rh-DOPE, and NBD-DOPE) dissolved in chloroform were evaporated in a glass test tube. Then, 60 mM phospholipid solution was prepared from the lipid film dissolved in *n*-decane solution. All vesicle formations were conducted by 60 mM phospholipid solution. Double planar asymmetric lipid bilayers were formed using the droplet contact method. First, 4 μL of DOPC solution containing 0.02 mol% Rh-DOPE was added into the first well, 2 μL of DOPC/DOPS (1 : 1 mol ratio) solution was added into the second well, and 4 μL of DOPC solution containing 0.02 mol% 7-nitro-2-1,3-benzoxadiazol-4-yl (NBD)–DOPE was added into the third well. Next, 19 μL of phosphate-buffered saline (PBS; 10 mM Na_2_HPO_4_/NaH_2_PO_4_, 137 mM NaCl, and 2.7 mM KCl; pH 7.4) containing 500 mM glucose was added to the first well. Eight microliters and 19 μL of PBS containing 500 mM sucrose were added into the second and third wells, respectively. A liquid dispensing system was used to control the application time (3.2 ms) and the pressure of the pulsed jet flow (300 kPa) (SJVC3000; Sanei Tech, Japan). The asymmetric vesicles-in-a-vesicle system was observed by CLSM (FV-1200; Olympus, Tokyo, Japan) with an oil-immersion lens (×60) using a diode laser (559 nm) for rhodamine (570–670 nm) and a diode laser (473 nm) for NBD (485–554 nm).

### NBD quenching assay

An asymmetric vesicles-in-a-vesicle system containing 0.02 mol% NBD–DOPE in the outer leaflet of a giant vesicle was prepared using the asymmetry assay. Eight microliters of the vesicle solution collected from the vesicle-generation well was added to 40 μL of PBS containing 500 mM glucose. Then, 2 μL of 80 mM sodium hydrosulfite was added to the outer solution of asymmetric vesicles. The generated asymmetric vesicles-in-a-vesicle system was observed by CLSM with an oil-immersion lens (×60) using a diode laser (473 nm) for NBD (485–554 nm). Fluorescence intensities were measured using image J (NIH, USA).

### Annexin V assay

Asymmetric vesicles-in-a-vesicle systems were prepared using lipid solutions of (i) DOPC (1st and 3rd wells) and DOPS/DOPE (9 : 1 mol%) (2nd well) or (ii) DOPC (2nd well) and DOPS/DOPE (9 : 1 mol%) (1st and 3rd wells). Next, 19 μL of HEPES buffer (10 mM HEPES and 140 mM NaCl; pH 7.4) containing 500 mM glucose was added to the first well. Six microliters of HEPES buffer containing 500 mM sucrose, 1 μL of 5-fold diluted Alexa Fluor 488-conjugated Annexin V solution, and 1 μL of 8 mM calcium ions were added to the second well. Nineteen microliters of HEPES buffer containing 500 mM sucrose was added to the third well. The asymmetric vesicles-in-a-vesicle was then generated by the pulsed jet flow. Alexa Fluor 488 in the asymmetric vesicles-in-a-vesicle was observed by CLSM with an oil-immersion lens (×60) using a diode laser (473 nm) for Alexa Fluor 488 (485–554 nm). Fluorescence intensities were measured using image J (NIH, USA).

### Observation of fusion of small vesicles into the giant vesicle

An asymmetric vesicles-in-a-vesicle system was prepared using lipid solutions of DOPC (3rd well), DOPC containing 0.02 mol% Rh-DOPE (1st well), and DOPC/DOPS/DOPE (4 : 5 : 1 mol%) or DOPC/DOPS (1 : 1 mol%) (2nd well). Next, 19 μL of HEPES buffer (10 mM HEPES and 140 mM NaCl; pH 7.4) containing 500 mM glucose was added to the first well. Eight microliters and 19 μL of HEPES buffer containing 500 mM sucrose were added to the second and third wells, respectively. The asymmetric vesicles-in-a-vesicle was then generated using pulsed jet flow. Eight microliters of vesicle solution collected from the vesicle-generation well was added to 36 μL of HEPES buffer containing 500 mM glucose. Then, 1 μL of 30 μM α-hemolysin and 5 μL of 50 mM CaCl_2_ were added to the outer solution of the asymmetric vesicles. The fusion of asymmetric vesicles-in-a-vesicle was observed by CLSM with an oil-immersion lens (×60) using a diode laser (559 nm) for rhodamine (570–670 nm).

## Results and discussion

### Formation of the vesicles-in-a-vesicle system

We previously reported that the size distribution of giant lipid vesicles formed using the pulsed jet-flow method could be regulated by changing the area of the planar lipid bilayer and the application time of the jet flow.^13^ For instance, when the area of the planar lipid bilayer is increased, the vesicle diameter increases. When the application time of the jet flow is increased, the vesicle diameter decreases. Based on these results, to generate giant vesicles containing smaller vesicles, that emulate asymmetric lipid distribution in the plasma membrane and intracellular vesicle membrane in eukaryotic cells, we developed a triple-well device that mounts two separators with apertures of diameter 100 μm (to generate inner vesicles) between the first and second wells and 500 μm in diameter (to generate cell-sized vesicles) between the second and third wells (Fig. 1(a) and (b)). The lipid monolayer of the second well in the triple well-device was shared by the outer leaflet of the inner vesicles and the inner leaflet of the giant vesicles. The asymmetric lipid distribution in the vesicle membrane is a crucial feature of the triple device.

The giant vesicles containing the smaller vesicles were generated by adjusting the width of the second well and the application time and pressure of the jet flow. When the vesicles-in-a-vesicle system was generated using the triple-well device with the width of the second well of 1.8 mm, the giant vesicles containing the smaller vesicles were not observed. We determined that the optimal width of the second well is 1.2 mm, application time of the jet flow is 3.2 ms, and pressure of the jet flow is 300 kPa. The results suggested that the vesicles-in-a-vesicle system was generated from each planar lipid bilayer. Under the abovementioned conditions (*i.e.*, jet flow application time: 3.2 ms and application pressure: 300 kPa), the generation ratio of cell-sized vesicles containing the smaller vesicles was approximately 18% (5/28, three explements). The generation ratio of the vesicles-in-a-vesicle system can be improved by modifying the size of the planar lipid bilayer, diameter of the jet nozzle, and pressure of the jet flow. The average diameter of the giant vesicles was 23.6 ± 9.2 μm. On the contrary, the diameter range of the inner vesicles within the giant vesicles was approximately 200–1000 nm.

### Asymmetry of the vesicles-in-a-vesicle system

**Fig. 2:**
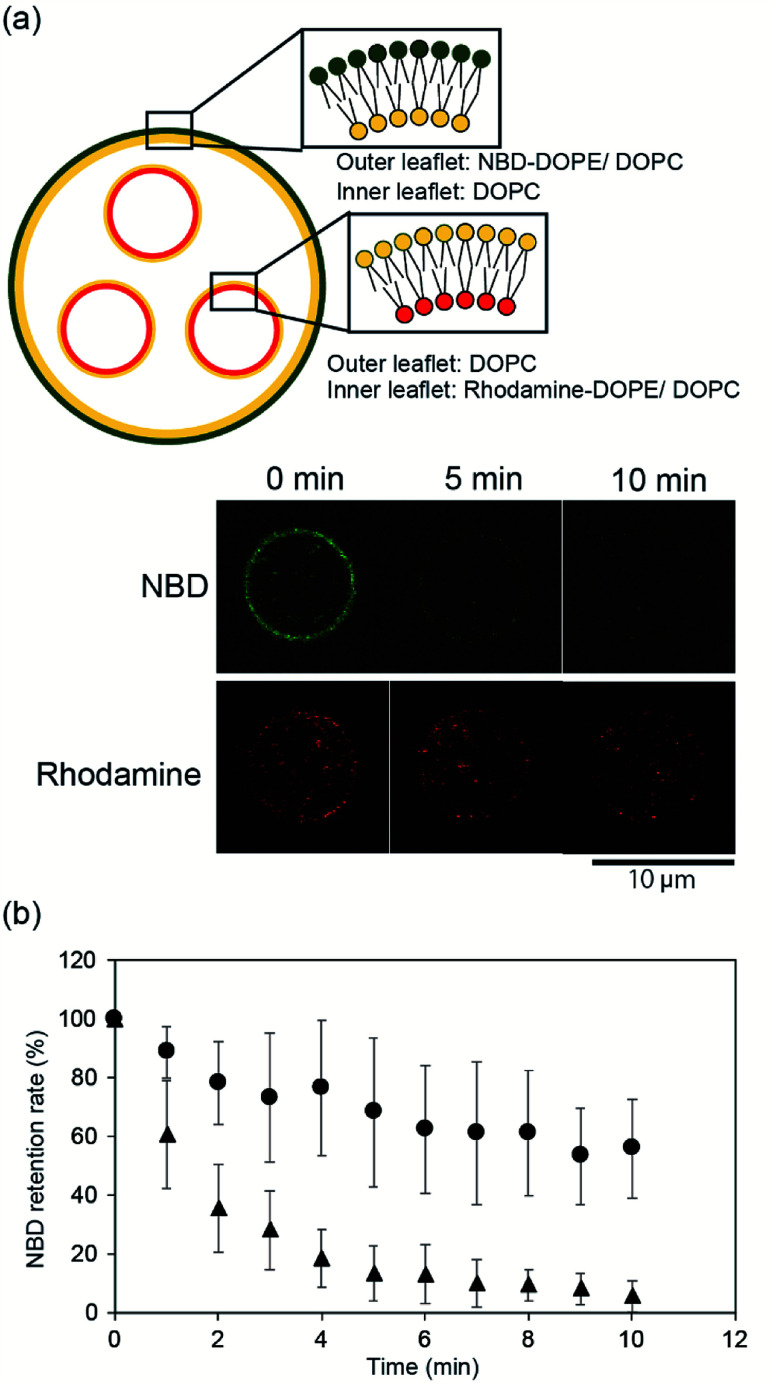
(a) Schematic representation of components of the asymmetric vesicles-in-a-vesicle system used for the fluorescence-quenching assay. The vesicles-in-a-vesicle system consisted of NBD-conjugated lipids in the outer leaflet of the giant vesicle. Typical time-lapse images of the vesicles-in-a-vesicle system obtained using the fluorescence-quenching assay. (b) Fluorescence intensities determined using the NBD-quenching assay. The graph shows the time-lapse of the NBD-quenching ratio of the asymmetric vesicles-in-a-vesicle (triangles) (*n* = 13) and symmetric vesicles-in-a-vesicle system with NBD-lipids on both leaflets (circles) (*n* = 10). Error bars, standard deviations.

To confirm the asymmetry of the giant vesicle membranes containing the smaller vesicles, we conducted fluorescence quenching of phospholipid-conjugated NBD in the outer leaflet of the giant vesicles. Sodium hydrosulfite was added to the outer solution of the giant vesicles. NBD in the outer leaflet of the vesicles was only quenched by sodium hydrosulfite.^15^Fig. 2(a) shows typical confocal microscopy images of the smaller vesicle-containing asymmetric giant vesicles, generated from the second planar lipid bilayer with 0.02 mol% NBD-conjugated DOPE/DOPC in the outer leaflet, before and after the addition of sodium hydrosulfite. NBD fluorescence on the giant vesicle membranes significantly decreased within 10 min. The retention rate of NBD fluorescence was approximately 5.5% ± 5.3%. The retention rate of NBD fluorescence on the symmetric giant vesicles was approximately 55.9% ± 16.9% (Fig. 2(b)). These results suggest that our device can be used to generate giant vesicles containing small vesicles with asymmetric lipid membranes.

**Fig. 3:**
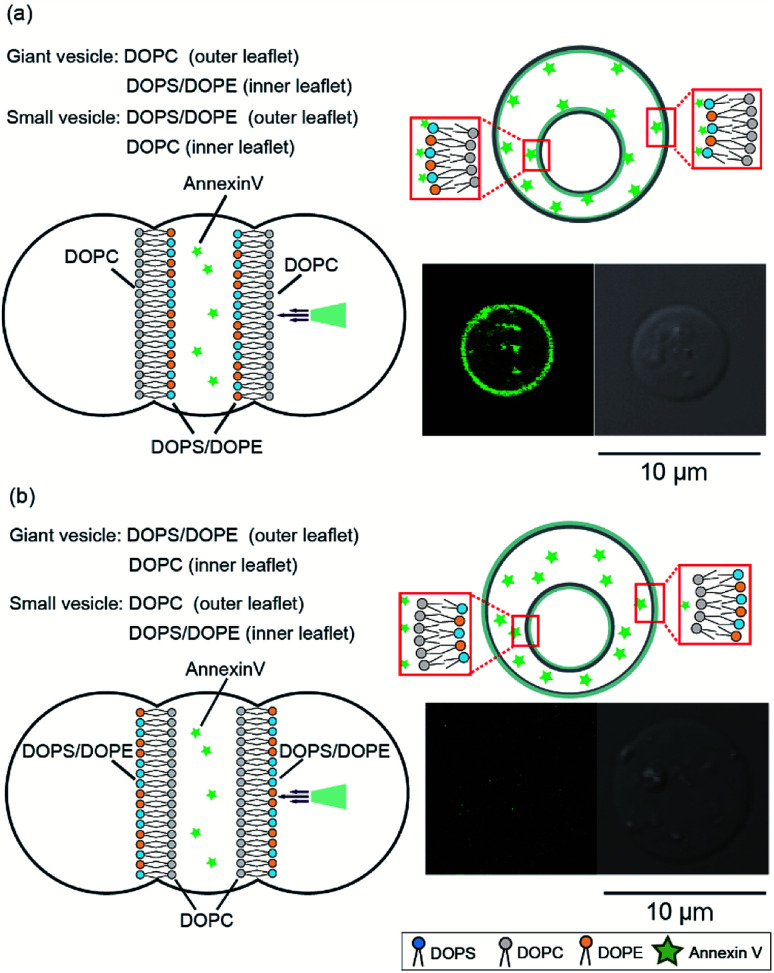
Investigation of asymmetry of the inner leaflet of the giant vesicles and the outer leaflet of the inner vesicles using fluorescent Annexin V. (a) Asymmetric vesicles-in-a-vesicle system composed of DOPC in the outer leaflet and DOPS/DOPE (9 : 1 mol%) in the inner leaflet of the giant vesicles and of DOPS/DOPE (9 : 1 mol%) in the outer leaflet and DOPC in the inner leaflet of the inner vesicles. (b) Asymmetric vesicles-in-a-vesicle composed of DOPS/DOPE (9 : 1 mol%) in the outer leaflet and DOPC in the inner leaflet of the giant vesicles and of DOPC in the outer leaflet and DOPS/DOPE (9 : 1 mol%) in the inner leaflet of the inner vesicles.

Next, we investigated the asymmetry of the inner leaflet of the giant vesicles and the outer leaflet of the inner vesicles using the fluorescent Annexin V binding assay. Annexin V was specifically bound to PS in the presence of calcium ions.^16^ We added Annexin V and calcium chloride to the second well containing the asymmetric vesicles-in-a-vesicle system. We generated giant asymmetric vesicles containing smaller vesicles using the following two methods: (i) DOPC solution was added to the first and third wells and DOPS/DOPE (9 : 1 mol%) solution was added to the second well and (ii) DOPC solution was added to the second well and DOPS/DOPE (9 : 1 mol%) solution was added to the first and third wells. Consequently, we obtained (i) giant asymmetric vesicles encapsulating smaller asymmetric vesicles composed of DOPS/DOPE (9 : 1 mol%) in the outer leaflet and DOPC in the inner leaflet and (ii) giant asymmetric vesicles and small vesicles with opposite compositions, respectively. Fig. 3 shows the schematics and typical confocal microscopy images of the giant asymmetric vesicles containing smaller asymmetric vesicles with the lipid compositions (i) and (ii). Annexin V fluorescence was observed on the giant and inner vesicle membranes composed of DOPC in the outer leaflet of the giant vesicles and the inner leaflet of inner vesicles, and of DOPS/DOPE (9 : 1 mol%) in the inner leaflet of giant vesicles and the outer leaflet of inner vesicles (lipid composition (i)) (Fig. 3(a)). Annexin V fluorescence on the asymmetric giant vesicles containing the smaller vesicles with lipid composition (ii) was not observed (Fig. 3(b)). The detection ratio of Annexin V-incorporated fluorescence on both giant vesicle membranes and inner vesicle membranes was 72.4%. Therefore, using this system, we generated giant asymmetric vesicles containing asymmetric vesicles to mimic asymmetric lipid distribution of eukaryotic cells for first time.^6^

### Fusion of the inner vesicles in the asymmetric vesicles-in-a-vesicle system

**Fig. 4:**
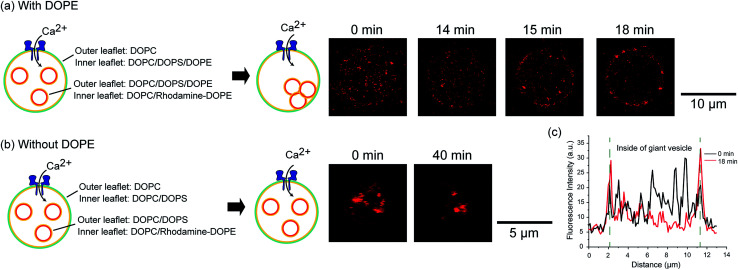
Demonstration of vesicular fusion of intracellular vesicles using the asymmetric vesicles-in-a-vesicle system. (a) Asymmetric vesicles-in-a-vesicle system with DOPE in the inner leaflet of the giant vesicles and the outer leaflet of the inner vesicles. (b) Asymmetric vesicles-in-a-vesicle system without DOPE. (c) Line profiles of rhodamine fluorescence intensity of asymmetric vesicles-in-a-vesicle system with DOPE in the inner leaflet of the giant vesicles and the outer leaflet of the inner vesicles. The black and red lines show the rhodamine fluorescence before and after calcium ion influx, respectively. The green-dashed lines represent the surface of giant vesicles.

We constructed an induction system for artificial exocytosis by calcium ion influx into giant vesicles. We prepared an asymmetric vesicles-in-a-vesicle system; the giant asymmetric vesicles were composed of DOPC in the outer leaflet and DOPC/DOPS/DOPE (4 : 5 : 1 mol%) in the inner leaflet, and the inner asymmetric vesicles were composed of DOPC/DOPS/DOPE in the outer leaflet and DOPC containing 0.02 mol% Rh-DOPE in the inner leaflet. The fusion of giant asymmetric vesicles and inner asymmetric vesicles was induced by calcium ion influx through nanopores into asymmetric giant vesicles. We added 0.6 μM (final concentration) nanopores (α-hemolysin) and 5 mM (final concentration) calcium ions to the outer solution of asymmetric giant vesicles. After approximately 15–20 min, we observed rhodamine fluorescence on the asymmetric giant vesicle membranes (Fig. 4(a)). The fluorescence of the inner rhodamine-vesicles was decreased in the asymmetric giant vesicles, while the rhodamine fluorescence intensity was increased on the giant vesicle surfaces (Fig. 4(c)). This result indicated that the inner rhodamine vesicles were fused to the membranes of the asymmetric giant vesicles. On the contrary, the fusion phenomenon of an asymmetric vesicles-in-a vesicle without DOPE (DOPC/DOPS (1 : 1 mol%)) in the inner leaflet of the giant vesicle and outer leaflet of the inner vesicles was not observed (Fig. 4(b)). This result indicated that the presence of DOPE might contribute to the promotion of vesicle fusion. The extracellular transport of the biomolecules *via* the inner (intracellular) vesicles into the biological cells was caused by membrane fusion between the intracellular vesicle membrane and plasma membrane in the presence of calcium ions, PS, PE and the membrane protein soluble *N*-ethylmaleimide-sensitive factor attachment protein receptors (SNARE).^17^ Our exocytosis system emulates the exocytosis system of eukaryotic cells in terms of membrane fusion induced by calcium ions and asymmetric lipid distribution.

## Conclusions

Although the fusion of symmetric lipid vesicles has been reported,^9,10^ this is the first observation of fusion between giant asymmetric vesicles and asymmetric vesicles within giant asymmetric vesicles. This vesicle fusion system, which mimics the fusion phenomenon of intracellular vesicles, will be useful for investigating models of intracellular vesicular traffic, such as autophagy or exosomes. In artificial cell studies, a bioreactor for releasing medicines by external input, such as ions, heat, and light, can be developed using this vesicles-in-a-vesicle system in the future. Moreover, the use of this asymmetric vesicles-in-a-vesicle system will help understanding of mechanisms of vesicle transport, such as neurotransmission and exocytosis.

## Conflicts of interest

There are no conflicts to declare.

## Supplementary Material
